# Fatal Case of *Plasmodium vivax* Malaria in a Splenectomized Patient

**Published:** 2012

**Authors:** H Mahmoudvand, L Farivar, I Sharifi, M Fasihi Harandi, V Moazed, S Jahanbakhsh, Z Babaei, N Zia-Ali

**Affiliations:** 1 Department of Medical Parasitology and Mycology, Kerman University of Medical Sciences, Kerman, Iran; 2 Department of Medical Parasitology and Mycology, Tehran University of Medical Sciences, Tehran, Iran; 3 Leishmaniasis Research Center, Kerman University of Medical Sciences, Kerman, Iran; 4 Department of Medical Oncology and Hematology, Kerman University of Medical Sciences, Kerman, Iran

**Keywords:** *Plasmodium vivax*, Malaria, Splenectomy, Iran

## Abstract

Malaria is a major problem in tropical and sub-tropical countries, with high morbidity and mortality. Splenectomy makes patients more susceptible to serious bacterial and parasitic infections. We report for the first time in Iran a fatal case of *Plasmodium vivax* malaria, confirmed by microscopic and molecular (Semi-nested multiplex PCR) tests in a patient who had undergone splenectomy due to hemolytic anemia.

## Introduction

Malaria is a major problem in tropical and sub-tropical countries, accounting for about 216 millions episodes and 665000 malaria deaths reported in 2010 (1, 2). It is estimated that up to 40% of the world's population is at risk for infection with malaria. The most serious clinical forms are caused by *Plasmodium falciparum and P. vivax* (2).

The role of the spleen in elimination of intra-erythrocytic parasites has been reported in several animal models. Splenectomy is increasingly practiced in malaria-endemic tropical countries. The procedure leaves patients more susceptible to parasitic infections, including malaria (3).

Here, we report for the first time in Iran a fatal case of *P. vivax* malaria in a patient who had previously undergone splenectomy caused by hemolytic anemia.

## Case report

A 40-year-old man from Sistan-Baluchistan Province, the main endemic focus of malaria in Iran (4) was admitted to Bahonar Hospital in Kerman in September 2010. He had previously undergone splenectomy due to hemolytic anemia. The symptoms subsided after the surgery, but four months later he developed anemia, jaundice, malaise, fever and weakness, for which he was admitted to Bahonar Hospital in the city of Kerman.

Upon admission his blood pressure was 100/60 mmHg, pulse rate 100/min, respiration rate 20/min, body temperature 39^0^C and he appeared pale and icteric.

Laboratory findings included the following; hemoglobin level, 8 g/dl; platelet count 43000 platelets/mm^3^; erythrocyte sedimentation rate 35 mm/h; and total serum bilirubin 3.7 mg/dl. His prothrombin time (PT) was 16 seconds and partial thromboplastin time (PTT) was 42 seconds, with a noticeable increase (according to normal range PT: 12s and PTT: 24-36s).

The VDRL and PPD tests were negative. In addition, direct agglutination test (DAT) for detection of visceral leishmaniasis (kala-azar), blood and urine culture for bacterial growth were negative. Also rheumatoid factor and negative C-reactive protein (CRP) were negative but a week later CRP was positive. The direct /indirect antiglobulin tests (DAGT/IAGT) were negative but the liver function tests were more than normal limits. No abnormality was detected on chest X-ray and abdominal CT scan showed hepatomegaly, but no lymphadenopathy was observed.. While cerebrospinal fluid examinations were normal. There was no evidence of underlying disorders and diagnostic tests for human immunodeficiency virus (HIV), hepatitis B and C viruses were negative.

From the first day of admission, suspicion of bacterial infection lead to treatment with antibiotics such as; ceftriaxone, vancomycin and imipenem in the recommended doses for two weeks, he also received pack cell and fresh frozen plasma to improve his blood indices.

**Fig. 1:**
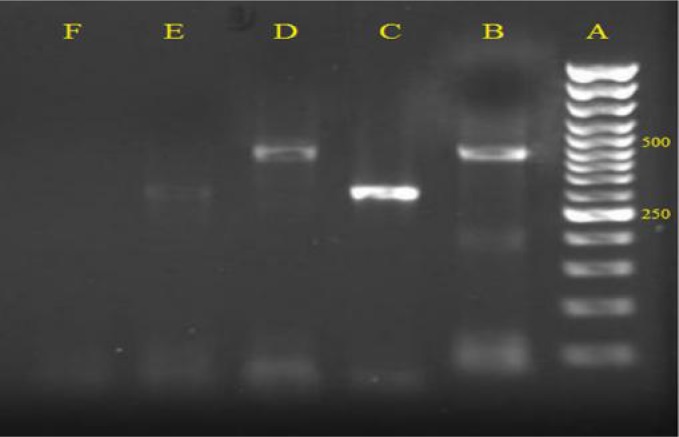
Lane A, DNA size marker 50 bp; Lane B, *Plasmodium vivax* (positive control, 499 bp); Lanes C *and E Plasmodium falciparum* (positive control, 395 bp); Lane D, *Plasmodium vivax* obtained from the peripheral blood smear preparation of the patient and Lanes F, negative controls

On the third day of hospital admission peripheral blood smears were examined for presence of malaria parasites for several times, but no malaria parasite was detected. On day 17 the patient's peripheral blood smear (PBS) was sent to the Department of Medical Parasitology and Mycology, at the School of Medicine, Kerman University of Medical Sciences. Examination of blood films showed asexual forms of *P. vivax*, however neither sexual forms nor *P. falciparum* stages were observed. On day 18, the patient received oral chloroquine phosphate (600 mg at 0 hours, 300 mg at 6, 24, 48 hours) and then due to the critical condition of the patient he received intramuscular artesunate two times per day for six days and one dose of oral fansidar (24 mg/kg). Unfortunately, after antimalaria treatment on day 24 his clinical state became deteriorated. He was transferred to the intensive care unit (ICU), subsequently, on day 30 he lost consciousness, with convulsions and developed signs of brain involvement and eventually went to coma and died. After 2 weeks, Semi-nested multiplex PCR based on the sequence of the small subunit ribosomal RNA (ssrRNA) gene was performed according to the method of Edoh et al. (5) and Rubio et al. (6), that confirmed the presence of *P. vivax* in blood smear of the patient (Fig. 1), also the amplicon was sequenced and recorded in the GenBank under the following Accession Number: JN084167.

## Discussion

Several clinical syndromes are caused by protozoan parasites of the genus *Plasmodium*. The most serious forms are caused mainly by *P. falciparum* and to a lower extent *P. vivax* (1). Splenectomy increases susceptibility and severity of malaria that can be very serious and the most important reason for fatality (3). Clinical manifestations of malaria are numerous including; fever, anemia, circulatory and immunopathologic changes as the result of erythrocytic invasion by the species of *Plasmodium*.

Though severe complication is rarely reported by *P. vivax* infection in which coma, sudden death or other symptoms of cerebral involvement have been observed (7–10). Kochar, et al. (11) have concluded that *P. vivax* can also be a cause of both sequestration and non-sequestration associated complication of severe malaria commonly involving the clinical manifestations of *P. falciparum* and rarely *P. vivax* that provoke changes in cerebral site and coma. However there are not any evidences to associate the fatal outcome of the patient with cerebral involvement caused by *P. vivax*.

The case in our report was a patient who was admitted to the hospital with severe anemia. After undergoing splenectomy, the clinical signs subsided intermittently but later on a more complicated clinical profile emerged as characterized by severe fever. There was no evidence of bacterial and viral infections and underlying disorders also serological tests for visceral leishmaniasis were negative. In spite of antibiotic therapy for two consecutive weeks, clinical condition did not improve. Microscopic and molecular tests on blood smear of the patient, confirmed the presence of *P. vivax*.

Eventually the patient received antimalarial drugs several days after admission, when serious clinical symptoms were developed. The patient did not respond to anti-malarial chemotherapy and eventually died after a phase of comatose and unconsciousness.

It is recommended that prophylactic measures should be taken, when splenectomized patients living in high risk malarious areas. In addition, early diagnosis and prompt treatment of such patients require special attention.
